# Development and Psychometric Validation of the Chinese Online Disinhibition Scale for Adolescents

**DOI:** 10.1002/pchj.70117

**Published:** 2026-08-11

**Authors:** Ziyao Zhou, Yujun Ma, Lin Wang

**Affiliations:** ^1^ Ginling College Nanjing Normal University Nanjing China; ^2^ Department of Social Work The Chinese University of Hong Kong Shatin Hong Kong; ^3^ Department of Social Work Fudan University Shanghai China

**Keywords:** adolescent, cross validation, online disinhibition, online disinhibition effect

## Abstract

Online disinhibition significantly influences adolescents' communication and behaviors in digital environments, yet culturally adapted measurement tools remain limited in China. This study aimed to develop and validate a Chinese version of the Online Disinhibition Scale (ODS‐C) based on Suler's (2004) theoretical framework. We translated and adapted Cheung et al.'s (2020) questionnaire through rigorous forward and backward translation protocols. Using a calibration sample (*n* = 686), we tested the scale's internal structure, reliability, and construct validity. A cross‐validation sample (*n* = 687) was subsequently employed to verify and select the optimal internal structure of the ODS‐C, assess measurement invariance across gender, and examine criterion validity based on relationships with internet addiction and cyberbullying perpetration. The final ODS‐C demonstrated a hierarchical five‐factor structure, in which dissociative anonymity and invisibility functioned as two distinct first‐order factors subsumed under a higher‐order anonymity factor, while the remaining four dimensions—asynchronicity, solipsistic introjection, dissociative imagination, and minimization of authority—operated as independent first‐order factors. Results indicate that the ODS‐C demonstrates high internal consistency, robust construct validity, and significant criterion validity. Evidence of measurement invariance confirmed the stability of the factor structure, loadings, and variances across genders. The ODS‐C provides researchers and practitioners with a reliable and valid tool for assessing online disinhibition among Chinese adolescents, facilitating more culturally informed interventions and policies addressing digital behavior.

## 1 Introduction

In contemporary digital environments, adolescents increasingly navigate social connections through technology‐mediated interactions, transforming how they establish and maintain relationships with peers and communities. They rely on communication applications and social media platforms to engage with the world, seek connection, gain recognition and support, and develop their identities (Cross et al. 2015). Research has consistently demonstrated that online disinhibition significantly shapes their communication patterns and behaviors in digital environments (Chu et al. 2023; Stuart and Scott 2021; Udris 2014; Wang et al. 2024). Online disinhibition involves a loosening of social constraints in online interactions, leading to reduced concern for self‐presentation and diminished consideration of behavioral consequences (Suler 2004; Wright et al. 2019). Under these conditions, adolescents may express thoughts and exhibit behaviors online that they would typically suppress in face‐to‐face encounters, substantially affecting their social interactions and psychological wellbeing (Bargh et al. 2002; Liao et al. 2024; Wu et al. 2017).

Empirical evidence has indicated that online disinhibition exerts considerable influence on adolescents. Some studies have shown that the negative manifestations of online disinhibition have been linked to heightened risks among adolescents, including cyberbullying perpetration and victimization, deviant behaviors, and online hate activities (Liao et al. 2024; Wachs et al. 2019; Wang et al. 2024; Yang et al. 2021). Moreover, these relationships are often mediated by cognitive mechanisms such as moral disengagement and deficits in self‐control (Wang et al. 2022). These adverse associations vary according to individual psychological traits, such as anxiety, low empathy, or callous‐unemotional features, which correlate with elevated disinhibition (Antoniadou et al. 2016; Green et al. 2016). The negative influence of online disinhibition also shows gender differences, whereby boys typically exhibit higher levels of disinhibition and more frequent negative behaviors (Huang et al. 2020). Beyond the negative outcomes of online disinhibition, others focused on positive interpretations of online disinhibition highlight enhanced self‐disclosure in anonymous contexts, emotional support, prosocial interactions, and improved social well‐being (Ko and Kuo 2009; Stuart and Scott 2021; Varnali and Toker 2015). This inherent complexity in empirical findings stems from differing conceptual explanations and operationalization of online disinhibition.

A literature review of quantitative empirical research on online disinhibition identifies three primary categories of measurement scales used in adolescent studies. Each of them reflects distinct theoretical assumptions and carries specific limitations that collectively underscore the need for a more integrative approach.

The first category conceptualizes online disinhibition as features of digital contexts, where adolescents experience reduced social restraints compared to offline settings (Yang et al. 2021). This approach predominantly examines how specific technological attributes, particularly anonymity, asynchronicity, and invisibility, predict behaviors, with a strong emphasis on harmful actions (Wang et al. 2022). Anonymity has been especially central, measured via dedicated scales such as the 4‐item Anonymity subscale (Barlett and Gentile 2012) or the 4‐item Perceived Anonymity scale (Jung et al. 2012), which serve as proxies for online disinhibition. To overcome single‐dimensional limitations, some studies employed composite scales integrating asynchronicity, invisibility, and anonymity to measure online disinhibition (Wang et al. 2020; Wang and Ngai 2020). However, this category has obvious limitations. For one thing, it focuses on objective online context features while neglecting individuals' subjective perceptions in this context (Cheung et al. 2020; Udris 2014). For another, cultural contexts are often overlooked, leading to inconsistent findings. For instance, anonymity and invisibility show no direct association with cyberbullying in Chinese contexts, whereas asynchronicity is a key predictor (Wang and Ngai 2020). In contrast, Western studies indicated that anonymity and invisibility directly foster online disinhibition and support social learning processes contributing to adolescent cyberbullying (Lowry et al. 2016). These discrepancies emphasize the need to incorporate cultural factors when examining links between online disinhibition and adolescent behaviors.

The second category adopts a dual‐outcome framework, most prominently represented by Udris's (2014) 11‐item Online Disinhibition Scale, which grounds its conceptualization in the distinction between toxic and benign manifestations of disinhibition originally proposed by Suler (2004). Benign disinhibition could foster kindness, support, self‐disclosure, and emotional expression (Lapidot‐Lefler and Barak 2015; Stuart and Scott 2021; Suler 2004), while toxic disinhibition may lead to harassment, verbal aggression, and online violence (Wachs et al. 2019; Wachs and Wright 2019; L. Wang and Ngai 2020). Research indicated that high levels of toxic online disinhibition increase adolescents' likelihood of engaging in harmful online behaviors (Huang et al. 2020; Wachs et al. 2019). Benign disinhibition has yielded mixed outcomes, and it is associated with higher levels of social well‐being and predicts positive online behaviors yet also correlates with increased cyberbullying victimization (Varnali and Toker 2015; Chu et al. 2023). Despite these advances, current measurement approaches remain limited. This binary categorization may obscure the context‐dependent and culturally variable nature of disinhibited behavior. What constitutes toxic disinhibition in one cultural setting or online subculture may be perceived as normative or even prosocial in another (Suler 2004). Furthermore, this framework does not account for the psychological mechanisms through which individuals arrive at disinhibited states, focusing instead on behavioral outcomes rather than the underlying perceptual processes (Clark‐Gordon et al. 2019; Stuart and Scott 2021).

The third category is represented by the 28‐item Measure of Online Disinhibition (MOD) developed by Stuart and Scott (2021), which conceptualizes online disinhibition as a psychological dynamic internal to the individual rather than a product of situational features. This scale operationalizes disinhibition through constructs such as disinhibited self‐disclosure, online self‐confidence, and a sense of social liberation in digital environments, thereby shifting analytical focus from what the online context affords to how individuals psychologically experience and respond to it. Research using this scale has indicated that individuals who perceive the online environment as a safe and protected space—rather than those who feel compelled to manage their self‐presentation—are more strongly associated with online disinhibition (Scott et al. 2022). Nevertheless, this approach carries a significant limitation. By separating individual perception from the contextual features that shape it, the scale implicitly treats all online users as equally influenced by the online environment. As evidence from the first category already demonstrates, different technological features, such as anonymity, asynchronicity, and invisibility, produce distinct psychological and behavioral outcomes. Therefore, this measurement may lose explanatory precision, especially when comparing results across various platforms or cultural contexts.

These three categories of measurement reveal a persistent tension between context‐centered and person‐centered approaches, and none fully integrates both dimensions within a unified psychometric instrument. Cheung et al. (2020) Online Disinhibition Scale (ODS) represents a more comprehensive attempt to bridge this gap, grounding measurement in Suler's (2004) six‐factor theoretical framework while simultaneously accounting for the interplay between Internet affordances and individuals' subjective perceptions of those affordances. There are six dimensions in Suler's (2004) theoretical framework and the ODS: dissociative anonymity (DA), invisibility (IV), asynchronicity (AS), solipsistic introjection (SI), dissociative imagination (DI), and minimization of authority (MA).

The six dimensions proposed by Suler (2004) do not operate as a simple checklist of independent predictors. Rather, they collectively instantiate a process of **psychological dissociation between the online self and the offline self** (Barak et al. 2008; Suler 2004). This dissociative process is the theoretical mechanism through which disinhibition emerges. When individuals perceive their online identity as detached from their real‐world self and remain invisible to others, the social cues that normally regulate face‐to‐face behavior lose their force. This reduces the anticipated costs of norm violation, lowering the psychological threshold for uninhibited expression. Asynchronicity further deepens this process by removing the immediacy of social feedback. Unlike synchronous face‐to‐face interaction, individuals act without seeing how others respond in asynchronous communication, so the immediate social feedback that normally sustains social inhibition is absent (Barak et al. 2008; Suler 2004). The remaining three dimensions operate at a deeper intrapsychic level. Solipsistic introjection describes how individuals mentally construct imagined versions of their online friends, internalizing the conversation as a kind of inner dialogue. This blurs the boundary between self and other, reducing the psychological distance that normally sustains interpersonal restraint. Dissociative imagination extends this boundary dissolution further. Individuals come to perceive the online world as a separate realm in which real‐world moral and social rules are felt to be suspended. Finally, minimization of authority removes the external regulatory anchor provided by hierarchical social structures. When the symbols and agents of authority are absent or perceived as ineffective, individuals experience fewer external constraints on their behavior. Taken together, these six dimensions converge on a common psychological outcome. That is the attenuation of the self‐monitoring, social accountability, and norm internalization processes that govern behavior in offline contexts. This theoretical coherence is what justifies treating these six dimensions as constituent components of a single construct rather than as unrelated situational variables (Suler 2004).

The ODS demonstrates strong psychometric properties, as evidenced by its reliability, convergent validity, and discriminant validity (Cheung et al. 2020). Despite the conceptual comprehensiveness of the ODS, its applicability across cultural contexts remains an open empirical question. This concern is particularly salient in the Chinese context, where distinctive regulatory frameworks and sociocultural norms substantially shape adolescents' online experiences in ways that may not be adequately captured by instruments developed and validated in Western settings (Wang et al. 2024). In China, the government has implemented special governance initiatives addressing negative online behaviors affecting minors' healthy development. These initiatives focus on rectifying online platforms that lack proper real‐name registration systems, fail to enforce adequate controls on minors' gaming time, and provide non‐compliant paid services (CINIC 2024). The government has also promoted anti‐addiction models for online streaming and video platforms while improving mechanisms such as real‐name authentication, function restrictions, time limits, content review, and algorithm recommendations. These regulatory measures directly affect individuals' sense of anonymity in online environments and adolescents' perception of authority regarding online behavior (Chu et al. 2023; Yang et al. 2023). Considering these specific cultural contexts and social norms is crucial to understanding the online disinhibition effect among Chinese youth and its behavioral consequences. Assessing the suitability of this scale for Chinese distinctive online context is necessary and highly informative, allowing for a comprehensive comparison of ODS in terms of similarities and differences across societies and cultures.

The present study aims to culturally adapt and psychometrically validate a Chinese version of the Online Disinhibition Scale (ODS‐C) for use with adolescents. Such an instrument is intended to capture the disinhibition effects manifested across behavioral and cognitive domains, providing a theoretically grounded and culturally sensitive tool for understanding adolescents' psychological states and behaviors in digital contexts (Joinson 2007; Suler 2004). Specifically, we translated and adapted the English scale developed by Cheung et al. (2020) within Suler's theoretical framework and tested its psychometric properties in a sample of Chinese adolescents.

To ensure the robustness of the scale's factor structure and avoid confirmation bias, we conducted an additional test of the revised model on a separate sample to evaluate its repeatability. Given that the present study employs the same theoretical framework and retains the core item content through systematic translation and cultural adaptation, the structural coherence of each subscale is expected to be preserved. What's more, prior research has documented gender differences in the *levels* of online disinhibition (Huang et al. 2020; Wachs et al. 2019). To examine whether the ODS captures the same underlying psychological constructs in the same way across gender groups, we conducted measurement invariance testing **to evaluate whether** online disinhibition operates as a universal psychological mechanism **equivalently** across gender groups. In addition, to demonstrate criterion validity, we examined the scale's associations with two behavioral outcomes: Internet addiction and cyberbullying perpetration. These variables were selected because they represent theoretically and empirically distinct manifestations of online disinhibition across different behavioral domains. Internet addiction reflects impaired self‐regulation in digital environments, a process that prior research has linked to heightened disinhibition (Liao et al. 2024). Cyberbullying perpetration represents another key behavioral manifestation of online disinhibition, reflecting its harmful interpersonal consequences and psychological impact on adolescents (Wachs et al. 2019; Wang et al. 2024). These two outcomes provide a comprehensive test of the scale's ability to capture the multidirectional consequences of online disinhibition.

To accomplish these purposes, we considered five hypotheses:

Hypothesis 1 *Online disinhibition is best represented by a six‐factor model with acceptable fit indices*.

Hypothesis 2 *The scale and subscales demonstrate high internal consistency*.

Hypothesis 3 *Each latent variable shows high shared variance (convergent validity) and is distinct from others (discriminant validity)*.

Hypothesis 4 *The scale shows robust invariance across genders*.

Hypothesis 5 *Online disinhibition dimensions positively correlate with Internet addiction and cyberbullying perpetration*.

## 2 Methods

### 2.1 Participants and Procedures

This study was conducted in four secondary schools in Xiangcheng City, Henan Province, China. Participants were selected using a multistage cluster sampling approach. First, 4 schools (Grades 7–8) were randomly selected from 18 eligible secondary schools in the research site. Next, two classes from each grade in the selected schools were randomly sampled, and all students in these classes were included in the study. Data collection took place during regular class sessions, where participants completed a 30‐min self‐administered paper‐and‐pencil questionnaire. Teachers and trained research assistants supervised the process to ensure order and clarity. Since participants were minors aged 11–16 years, written informed consent was obtained from the schools, parents, and students before the study commenced.

In determining the sample size, we adhered to a 10:1 subject‐to‐item ratio, which set a minimum requirement of 230 participants for the 23‐item measure. We also drew upon Comrey and Lee's (1992) recommendation, which rates sample sizes over 500 as “very good” for factor analysis. This approach ensured sufficient statistical power for the follow‐up analyses. The total sample consisted of 1373 students, including 761 boys (55.4%) and 612 girls (44.6%). Participants were enrolled in 7th grade (650 students, 47.4%) and 8th grade (723 students, 52.6%). Regarding household registration, 972 students (70.7%) were registered in rural areas, while 401 students (29.3%) were registered in urban areas. The mean age of the participants was 13 years (SD = 0.78). The mean age at which participants began using the Internet was 9 years (SD = 2.1). This study received ethical approval from the Survey and Behavioral Research Ethics Committee of the Chinese University of Hong Kong.

### 2.2 Measures

The Online Disinhibition Scale was developed by Cheung et al. (2020) based on Suler (2004) framework, which viewed online disinhibition as consisting of six dimensions: dissociative anonymity (DA), invisibility (IV), asynchronicity (AS), solipsistic introjection (SI), dissociative imagination (DI), and minimization of authority (MA). Each dimension focused on online behaviors, with a 7‐point Likert scale (*strongly disagree* to *strongly agree*) used for all 23 items. DA consists of 4 items, evaluating the extent to which an individual perceives the ability to conceal or alter their identity online. IV includes 5 items, assessing the perception of being unseen by others in the online environment. AS contains 3 items, measuring the perception that communication allows delayed responses online. SI has 3 items, exploring the extent to which individuals perceive a mental image or voice of others during online communication. DI involves 4 items, gauging the perception of the online environment as a separate, imaginary world disconnected from reality. MA comprises 4 items, assessing the perception of diminished influence from real‐life authorities in online spaces. The scale has demonstrated good psychometric properties.

This study translated and culturally adapted the Online Disinhibition Scale (ODS) originally developed by Cheung et al. (2020). For Online Disinhibition Scale's translation, we adhered to established guidelines for test translation (Van de Vijver and Hambleton 1996). First, a bilingual researcher translated the Online Disinhibition Scale from English into Chinese. The translated version was then translated back into English by a Chinese researcher proficient in English. A team of researchers subsequently reviewed the back‐translation and made slight adjustments to the wording to enhance its clarity and readability for Chinese‐speaking adolescents.

Internet addiction was measured using Young's 8‐item Internet Addiction Scale (Young 1998). It is a 5‐point scale ranging from 1 (*strongly disagree*) to 5 (*strongly agree*), where higher scores indicate greater severity of internet addiction (Young and De Abreu 2010). The Chinese version of this instrument has demonstrated robust psychometric properties and has been validated among adolescent populations in China (Lai et al. 2013; Wang et al. 2011). In this study, the scale showed strong internal consistency, with a Cronbach's alpha coefficient of 0.892.

Cyberbullying perpetration was measured with the European Cyberbullying Intervention Project Questionnaire (ECIPQ). Seven items were used for measuring cyber aggression, and each item was rated on a 5‐point scale (ranging from 0 = *never* to 4 = *always*). The Chinese version of the ECIPQ was developed by Zhu et al. (2022) and has exhibited good psychometric properties in studies on Chinese adolescent (Wang et al. 2025; Cai et al. 2025). In this study, the Cronbach's alpha of the scale is 0.901, demonstrating good reliability.

### 2.3 Data Analysis

The sample was randomly split into two groups using the Random Sample of Cases function in SPSS. Group 1 (*n* = 686) was used for exploratory factor analysis (EFA), while Group 2 (*n* = 687) was used for confirmatory factor analysis (CFA), assessment of psychometric properties, and tests of measurement invariance. The two groups are similar in demographic composition. The EFA group consisted of 56.6% boys, 48.5% seventh graders, with a mean age of 13 years. The CFA group included 54.3% boys, 46.1% seventh graders, and also had a mean age of 13 years.

EFA was conducted using SPSS 25, employing principal component analysis with varimax rotation. CFA was performed in Amos 23, utilizing maximum likelihood estimation with 2000 bootstrap samples to account for multivariate nonnormality (as indicated by Mardia's statistic > 5). The dimensions of online disinhibition were treated as latent variables, each measured by a set of manifest variables (the scale items). Model fit was assessed using multiple criteria: *χ* ^2^/df ratio (< 3 optimal, 3–6 acceptable), comparative fit index (CFI > 0.90), Tucker–Lewis Index (TLI > 0.90), normed fit index (NFI > 0.90), root mean square error of approximation (RMSEA < 0.08), and standardized root mean square residual (SRMR < 0.05 optimal, < 0.08 acceptable) (Bentler and Bonett 1980; Kline 2023).

## 3 Results

### 3.1 Exploratory Factor Analysis

For Group 1 participants, an EFA was performed. The Bartlett's Test of Sphericity and Kaiser‐Meyer‐Olkin (KMO) measure confirmed the data's suitability for factor analysis (*χ* ^2^ = 10150.93, df = 253, *p* < 0.001, KMO = 0.895). Our analysis yielded a five‐factor structure, which is consistent with Cheung et al. (2020) EFA result, but different from Suler's six‐factor model. This discrepancy arose because the items measuring DA and IV are loaded into the same factor (Factor 1). This pattern has been documented in prior research. For instance, in Hollenbaugh and Everett (2013)'s study, anonymity encompassed both dissociative anonymity and invisibility.

The model collectively explained 69.91% of the cumulative variance. Most items loaded strongly onto their intended factors (see Table 1), except for Item 1, which showed a slightly higher loading on Factor 5 than on Factor 1. Overall, the factor loading pattern closely matched that reported in Cheung et al.'s (2020) study, confirming the structural consistency of the scale.

**TABLE 1:** Results of EFA among Group 1 (*n* = 686).

Item	Factor 1	Factor 2	Factor 3	Factor 4	Factor 5
1	**0.507**	0.096	0.012	0.134	**0.514**
2	**0.612**	−0.038	−0.067	0.145	0.520
3	**0.721**	0.042	−0.022	0.111	0.420
4	**0.822**	0.107	0.079	0.028	0.175
5	**0.857**	0.118	0.118	0.063	0.114
6	**0.761**	0.164	0.211	0.073	0.161
7	**0.880**	0.132	0.164	0.074	0.028
8	**0.830**	0.130	0.281	0.069	0.000
9	**0.808**	0.169	0.308	0.064	−0.020
10	0.239	0.060	**0.826**	0.158	0.172
11	0.194	0.112	**0.875**	0.091	0.148
12	0.221	0.105	**0.867**	0.066	0.140
13	0.142	0.150	0.339	−0.113	**0.726**
14	0.145	0.192	0.335	−0.060	**0.662**
15	0.087	0.181	0.038	0.191	**0.586**
16	0.089	0.135	0.023	**0.721**	0.090
17	0.008	0.037	−0.009	**0.816**	0.140
18	0.093	0.168	0.116	**0.784**	−0.131
19	0.134	0.178	0.155	**0.743**	0.052
20	0.169	**0.724**	0.103	0.250	0.063
21	0.109	**0.813**	0.060	0.075	0.259
22	0.109	**0.857**	0.068	0.061	0.172
23	0.166	**0.752**	0.088	0.203	0.033
Eigenvalue	8.106	2.746	2.04	1.799	1.388
% of variance	24.18	12.229	12.013	11.441	10.046

*Note:* Primary factor loadings for each item are shown in bold.

### 3.2 Confirmatory Factor Analysis

**FIGURE 1:**
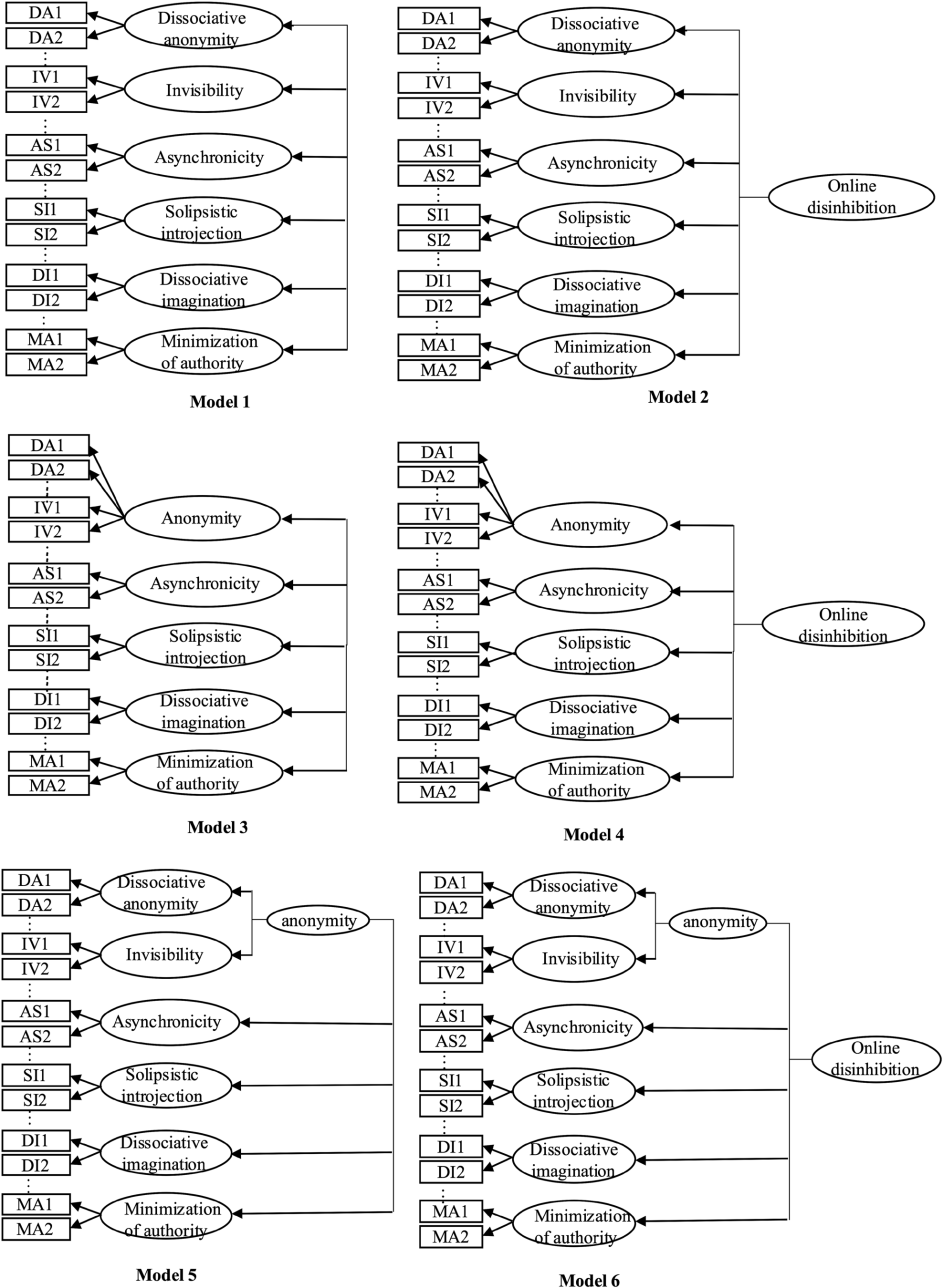
Six competing models.

For Group 2, we evaluated six competing models (Figure 1), following the framework proposed by Cheung et al. (2020). Models 1 and 2 assigned the 23 items to six correlated factors, aligning with Suler's (2004) theoretical framework, but with different hierarchical structures (Model 1 first‐order structure, Model 2 second‐order structure). Based on the EFA findings, Models 3 (first‐order structure) and 4 (second‐order structure) combined dissociative anonymity and invisibility into a single first‐order factor (anonymity), resulting in five first‐order factors. In Model 5, the 23 items were specified to load onto five correlated factors: four first‐order constructs and one second‐order factor (anonymity). This model examined whether the covariance between the two first‐order factors (DA and IV) could be explained by a higher‐order factor, suggesting that these two dimensions share sufficient common variance to reflect a common underlying construct. Model 6 proposed an alternative hierarchical model with one second‐order factor (anonymity) and four first‐order factors (AS, SI, DI, and MA).

Table 2 showed model fit tests. Most fit indices (CFI, TLI, NFI, SRMR, RMSEA) for Models 1–6 met recommended thresholds. However, Models 2, 3, and 4 exhibited excessively high *χ* ^2^/df ratios, while Models 1 and 5 demonstrated superior fit across multiple indices, making them viable representations of the online disinhibition scale's factor structure.

**TABLE 2:** Model fit indices of the 6 competing models (*n* = 687).

	Models					
	1	2	3	4	5	6
*X* ^2^	745.56	928.38	1053.22	1173.08	756.46	873.17
d.f.	215	224	220	225	218	223
*X* ^2^/df	3.47	4.14	4.79	5.21	3.47	3.92
CFI	0.95	0.93	0.92	0.91	0.95	0.94
TLI	0.94	0.92	0.91	0.90	0.94	0.93
NFI	0.93	0.91	0.9	0.89	0.93	0.92
SRMR	0.04	0.07	0.05	0.07	0.04	0.06
RMSEA	0.06	0.07	0.07	0.08	0.06	0.06
AIC	913.56	1078.38	1211.22	1078.38	918.46	1025.17

As noted by Stewart and Segars (2002), higher‐order factors provide a more parsimonious explanation for covariations among first‐order factors. Although Model 1 (first order) showed marginally better fit indices than Model 5 (higher‐order), the target coefficient (T) (Marsh and Hocevar 1985) revealed that the higher‐order factor accounted for over 90% of the covariation among the first‐order factors (T_1_/T_5_ = 745.56/756.46 = 0.98), supporting the validity of the more parsimonious structure. Thus, Model 5—despite its slightly weaker fit—offered a more economical representation of factor relationships and was selected for subsequent reliability and validity analyses.

### 3.3 Psychometric Properties

Following the assessment of overall model fit, we evaluated the scale's psychometric properties. The analysis revealed strong internal consistency, with a Cronbach's alpha of 0.92 for the full scale. Subscale reliability coefficients were as follows: 0.867 (DA), 0.944 (IV), 0.912 (AS), 0.799 (DI), 0.871 (MA), and 0.729 (SI).

All factor loadings demonstrated statistical significance and exceeded the 0.6 threshold, with the exception of item SI3 (detailed in Table 3). Composite reliability (CR) values surpassed 0.7 for all latent variables, confirming that the measurement items consistently represented their respective constructs.

**TABLE 3:** Results of CFA of Model 5 (*n* = 687).

	Item	Factor loading	SMC	*p*	CR	AVE
Dissociative anonymity	DA1	0.703	0.495		0.833	0.834
	DA2	0.789	0.623	***		
	DA3	0.828	0.686	***		
	DA4	0.823	0.677	***		
Invisibility	IV1	0.868	0.754		0.945	0.775
	IV2	0.835	0.697	***		
	IV3	0.921	0.849	***		
	IV4	0.895	0.8	***		
	IV5	0.881	0.776	***		
Asynchronicity	AS1	0.819	0.67		0.914	0.780
	AS2	0.913	0.833	***		
	AS3	0.913	0.834	***		
Dissociative imagination	DI1	0.646	0.418		0.806	0.511
	DI2	0.67	0.449	***		
	DI3	0.787	0.619	***		
	DI4	0.747	0.558	***		
Minimization of authority	MA1	0.731	0.534		0.873	0.634
	MA2	0.827	0.684	***		
	MA3	0.874	0.764	***		
	MA4	0.745	0.555	***		
Solipsistic introjection	SI1	0.851	0.724		0.753	0.518
	SI2	0.786	0.618	***		
	SI3	0.462	0.214	***		

***p < 0.001.

The validity assessment (Table 4) supported both convergent and discriminant validity. Convergent validity was established as all average variance extracted (AVE) values exceeded the recommended 0.50 threshold, indicating that the constructs accounted for more variance than measurement error. Discriminant validity was confirmed as the square root of each construct's AVE substantially exceeded all inter‐construct correlations.

**TABLE 4:** Results of the convergent and discriminant validity (*n* = 687).

	AVE	Anonymity	Asynchronicity	Dissociative imagination	Minimization of authority	Solipsistic introjection
Anonymity	0.823	0.906				
Asynchronicity	0.78	0.589	0.884			
Dissociative imagination	0.511	0.32	0.209	0.714		
Minimization of authority	0.634	0.438	0.289	0.487	0.796	
Solipsistic introjection	0.518	0.495	0.487	0.133	0.487	0.719

*Note:* diagonal shows the square roots of the AVE.

Criterion validity was estimated by correlating the scores of online disinhibition scale and the five subscales with participant's cyberbullying perpetration and internet addiction. As shown in Table 5, internet addiction was positively correlated with the total scores, and each subscale score for the online disinhibition. Cyberbullying behavior showed statistically significant but negligible correlations with overall online disinhibition and three of its five dimensions (*r*≈0.1). Correlations with anonymity and DI were non‐significant.

**TABLE 5:** Results of criterion validity (*n* = 687).

	Online disinhibition	Anonymity	Asynchronicity	Solipsistic introjection	Dissociative imagination	Minimization of authority
Internet addiction	0.381***	0.275***	0.209***	0.395***	0.155***	0.354***
Cyberbullying perpetration	0.103**	0.07	0.080*	0.080*	0.070	0.079*

*p < 0.05, **p < 0.01, ***p < 0.001.

### 3.4 Test of Measurement Invariance

Measurement invariance was tested to ensure the ODS‐C measures online disinhibition equivalently across genders. Given known gender differences in online disinhibition and digital behaviors (Helsper 2010; Huang et al. 2020; Niu et al. 2026; Wachs et al. 2019), this prerequisite confirms that any observed effects reflect true psychological differences rather than measurement artifacts. We tested measurement invariance through a series of multi‐group CFAs. The analysis followed a hierarchical approach (Table 6) progressively constraining parameters to evaluate configural (equal factor structure), metric (equal factor loadings), scalar (equal item intercepts), and strict invariance (equal residual variances). Model fit was assessed using the chi‐square difference test (Δ*χ* ^2^). complemented by practical fit indices (ΔCFI ≤ 0.01, ΔRMSEA ≤ 0.015, Chen, 2007) to minimize sensitivity to sample size. Results showed that the Δ*χ* ^2^ test was significant for the scalar invariance and strict invariance models, but the ΔCFI and ΔRMSEA values remained well within the accepted thresholds. Consequently, the results demonstrated full support for configural, metric, scalar, and strict invariance. This indicates that the scale's factor structure, factor loadings, intercepts, and residual variances are equivalent across genders.

**TABLE 6:** Results of test of measurement invariance (*n* = 687).

Model	df	χ^2^	RMSEA	CFI	SRMR	∆df	∆*χ* ^2^	∆RMSEA	∆CFI
Configural invariance	436	1122.555	0.048	0.935	0.05				
Metric invariance	453	1143.488	0.047	0.935	0.049	17	20.933	0.001	0.000
Scalar invariance	476	1188.803	0.047	0.933	0.049	23	45.315**	0.000	0.002
Strict Invariance	499	1249.849	0.047	0.929	0.487	23	61.046***	0.000	0.004

**p < 0.01, ***p < 0.001.

## 4 Discussion

The widespread and ubiquitous presence of internet‐connected devices in contemporary daily life has made it increasingly important to assess how digital environments impact adolescent behavioral and psychological outcomes. Recent literature has begun to focus on the online disinhibition effect, in which adolescents experience reduced social constraints and exhibit uninhibited behaviors online. The precise measurement of online disinhibition is essential for practitioners and researchers to enhance understanding of this phenomenon and its influence on individual psychological processes and behavioral outcomes. The current study developed a Chinese version of the Online Disinhibition Scale (ODS‐C) based on the theoretical dimensions of online disinhibition, specifically tailored for Chinese adolescents within their unique cultural and regulatory context (see Table 7).

**TABLE 7:** The Chinese Online Disinhibition Scale.

Dimension		Statement
Dissociative anonymity	Anonymity	我觉得我在网上是匿名的 I feel I am anonymous in the online environment
		我相信在网上别人并不知道我的个人身份 I believe that my personal identity remains unknown to others in the online environment
		我觉得我可以在网上隐藏自己是谁 I feel that I can hide my identity online
		我在网上的行为没人能认出来 My actions are not identifiable in the online environment
Invisibility		我感觉自己在网络环境中是隐身的 I feel like invisible in the online environment
		我觉得网络环境让别人看不到真正的我 I feel that online environment averts others from seeing any of me
		我在网上是隐身的 I am invisible online
		别人在网络环境中看不到我 Others do not see me in the online environment
		我的行为在网络环境中是隐形的 My actions are invisible in the online environment
Asynchronicity		在网上，我不需要立即回复别人 I do not need to reply others immediately in the online environment
		在网上，我可以延迟对他人的反馈 I can delay my feedback to others in the online environment
		在网上，我可以推迟给其他人回复 I can postpone replying others in the online environment
Solipsistic introjection		与别人在网络上交流时，我脑海中有他的形象或设想他的形象 I assign a character to that person I am communicating with online
		我在网上交流时根据自己的期望来解读别人的信息 I interpret others’ messages with my expectation during online communication
		我能理解网友/对方在网络环境中的表达 I perceive how that person's intended to talk about in the online environment
Dissociative imagination		我觉得网络空间里的人只是虚构的，与现实没有联系 I feel like people in the online space are just imaginary with no connection to reality
		网络环境是一个虚构的世界 The online environment is an imaginary world
		网络环境与现实没有任何联系 The online environment has no connection to reality
		线上环境与线下世界是分开的 The online environment is separated from the offline world
Minimization of authority		在网络环境中，我远离了现实生活中的权威 I am away from real life authorities in the online environment
		在网络环境中，我对现实生活中权威的恐惧感减弱了 I feel less fear of offline authorities in the online environment
		在网络环境中，我感觉自己不受现实生活中权威的束缚 I feel free from real life authorities in the online environment
		我感觉现实生活中的权威不存在于网络环境中 I feel that offline authorities are absent in the online environment

Our confirmatory factor analysis evaluated six competing models, ultimately selecting Model 5 to analyze the reliability and validity of factors and items. In Model 5, the 23 items loaded onto an alternative hierarchical structure comprising one second‐order factor (anonymity, encompassing first‐order factors DA and IV) and four first‐order factors (AS, DI, SI, MA). The empirical overlap between DA and IV observed in the present study is not entirely unexpected. At the theoretical level, these two dimensions are inherently interrelated, as feeling unidentifiable and feeling unseen in online environments are conceptually adjacent experiences that frequently co‐occur (Hollenbaugh and Everett 2013). Consistent with this, Cheung et al. (2020) similarly reported overlap between DA and IV items in their EFA, suggesting that this pattern reflects an inherent characteristic of the constructs rather than a sample‐specific artifact. Furthermore, Chinese real‐name registration policies in social media for internet users effectively reduce individuals' sense of dissociative anonymity, thereby sharpening the distinction between identity‐based and visual anonymity. Therefore, the final ODS‐C adopts a hierarchical five‐factor structure, comprising a higher‐order anonymity factor encompassing dissociative anonymity (DA) and invisibility (IV) as two distinct first‐order factors, alongside four independent first‐order factors: asynchronicity (AS), solipsistic introjection (SI), dissociative imagination (DI), and minimization of authority (MA).

Regarding the practical scoring of the ODS‐C, researchers may adopt different approaches depending on their research goals. At the subscale level, the scale yields **five‐dimension scores**: Anonymity, AS, SI, DI, MA, each scored by averaging its constituent items. For the anonymity dimension, DA and IV items are combined into a single score, reflecting their higher‐order unity within the hierarchical factor structure. This approach is particularly appropriate in the Chinese regulatory context, where the two subdimensions are structurally conflated and difficult to distinguish in practice. When research goals specifically require examining the differential effects of DA and IV, the two subdimensions may alternatively be scored separately.

This hierarchical model structure demonstrated appropriate fit for the Chinese adolescent sample under investigation. Most items loaded highly on their designated factors, indicating clear differentiation between the dimensions. Internal consistency coefficients (Cronbach's alpha) for each online disinhibition dimension were generally acceptable, ranging from 0.729 to 0.944, supporting our second hypothesis concerning reliability. Both the overall online disinhibition scale and its five subscales exhibited good reliability across two independent samples, demonstrating the robust nature of the instrument.

Regarding construct validity (Hypothesis 3), AVE values supported convergent validity, with all factors exceeding the recommended threshold of 0.50. Additionally, each factor's AVE exceeded the square of its correlations with other scale factors, confirming discriminant validity in the corrected sample. These findings indicate that each dimension of online disinhibition represents a distinct construct while remaining part of the broader online disinhibition framework.

The measurement invariance testing across gender (Hypothesis 4) demonstrated that the ODS‐C maintains consistent psychometric properties when applied to both boys and girls among the Chinese adolescents. This finding enhances the utility of the ODS‐C for research examining gender differences in online disinhibition, which some studies have considered boys may be more prone to experiencing toxic online disinhibition effects and bullying others on the Internet (Chu et al. 2023; Huang et al. 2020; Wachs and Wright 2019). The validation of measurement invariance across gender provides researchers using the ODS‐C with confidence that any observed differences reflect true variations in the underlying construct rather than measurement artifacts.

In criterion validity testing (Hypothesis 5), the results indicated a significant positive correlation between online disinhibition and cyberbullying perpetration. Given the very small effect sizes, these associations do not indicate a meaningful relationship, which is inconsistent with some previous research (Wachs et al. 2019). A possible explanation is that online disinhibition may function as a distal rather than proximal predictor of cyberbullying behaviors (Wang et al. 2024). For example, rather than directly motivating aggressive behavior toward others, DI may create a permissive psychological condition that facilitates cyberbullying (Suler 2004), and anonymity may operate through mediating cognitive mechanisms such as self‐regulation, self‐monitoring, and moral disengagement (Wang and Ngai 2020; Vernberg et al. 1999). The Chinese regulatory context may also help explain this finding. Due to real‐name registration requirements and enhanced governance of online violence, adolescents are increasingly aware that even in online environments, they may still face consequences for their negative behaviors, which may explain the lack of association between some online disinhibition factors and cyberbullying (Jiang 2016). Additionally, online disinhibition showed significant positive correlations with internet addiction, supporting our fifth hypothesis. This finding aligns with existing research suggesting that online disinhibition can lead to compulsive internet use through preference for online social interaction, subsequently correlating with internet addiction (Caplan 2010; Casale et al. 2015).

The current study makes significant contributions to the literature. First, it introduces the psychometrically validated Chinese instrument for measuring online disinhibition among adolescents. This study extends the application of online disinhibition assessment to Chinese cultural contexts, where internet use is governed by distinct regulatory frameworks and cultural norms. The scale can serve as a valuable tool for evaluating similarities and differences in online disinhibition across social, cultural, and media backgrounds, facilitating cross‐cultural research in this domain. Second, our research demonstrates the replicability of online disinhibition measurement in adolescent populations (ages 11–16) by establishing its reliability and validity across multiple samples. This age group represents a critical developmental period during which individuals establish online behavior patterns and identity development (Branje et al. 2021; Meeus et al. 1999), making accurate measurement particularly valuable for both research and intervention purposes.

Third, it provides an accurate measurement approach for understanding how internet characteristics and individual psychological traits co‐construct in the context of online disinhibition. Previous research has often focused predominantly on network characteristics, with limited attention to individual psychological processes under network conditions, particularly among adolescents, and how these psychological processes intertwined with network characteristics influence behavior (Wang and Ngai 2020; Wright et al. 2019; Yang et al. 2021). The present study addresses this limitation in two interconnected ways. For one thing, the ODS developed by Cheung et al. (2020) was chosen precisely because it operationalizes online disinhibition as the product of an interaction between digital affordances and individuals' subjective perceptions of those affordances, rather than treating either in isolation. Unlike context‐centered approaches that measure objective features as proxies for disinhibition, or person‐centered approaches that abstract away from situational context entirely, the ODS captures how individuals psychologically experience and interpret the affordances of their digital environment. For another, the cultural adaptation process itself, we argue that the specific sociocultural and regulatory context of Chinese adolescents meaningfully shapes how digital affordances are perceived and experienced. By systematically examining whether the ODS factor structure holds in this context, the present study moves beyond assuming a uniform relationship between digital features and individual perceptions, instead treating that relationship as culturally situated and empirically open to question.

Despite providing researchers with a useful scale, several limitations must be considered when interpreting our findings. First, this study narrowed its focus to middle school students, representing a critical period for educating young people about internet and media use. Additionally, the sample showed an imbalance between rural and urban registrations, possibly due to the site's moderate urbanization. Future research would benefit from extending investigations to more diverse populations in China and replicating the study across different cultural and geographical contexts to establish broader generalizability. Second, the present study only assessed correlations between online disinhibition and cyberbullying and internet addiction. While these outcomes are highly relevant, future research could examine relationships between online disinhibition and a broader range of outcomes. Third, while this study identified general characteristics of online disinhibition, group differences and mechanisms behind its benefits and risks remain incompletely understood. Future research should investigate potential moderators and mediators of the relationship between online disinhibition and behavioral outcomes, which could inform more targeted interventions.

Fourth, the cross‐sectional nature of our study prevents causal inferences regarding the relationships between online disinhibition and behavioral outcomes. Longitudinal studies are needed to determine predictors of online disinhibition and to examine how changes in online disinhibition over time relate to changes in behavioral outcomes. Future research should administer the ODS‐C at multiple time points to establish test–retest reliability coefficients and clarify the trait–state nature of the construct. Additionally, examining whether ODS‐C scores vary systematically across different digital platforms or social contexts would help determine the degree to which disinhibition reflects stable individual differences versus situational responses. Finally, our study relied exclusively on self‐report measures, which may be subject to social desirability bias, particularly when assessing socially undesirable behaviors such as cyberbullying. Future research could incorporate multiple assessment methods, including behavioral measures, peer reports, or digital trace data, to provide a more comprehensive assessment of online disinhibition and its behavioral correlates.

## 5 Conclusions

This study developed and validated a Chinese version of the Online Disinhibition Scale for adolescents, demonstrating robust psychometric properties including a clear five‐factor structure, high reliability, and good construct validity. The scale aligns with Suler's (2004) theoretical framework while accounting for the unique cultural and regulatory context of Chinese internet use. Our findings confirm the multidimensional nature of online disinhibition and its associations with problematic online behaviors including cyberbullying perpetration and internet addiction.

The scale will provide researchers with a measurement tool to understand the psychological processes underlying adolescent online behavior in the Chinese context. From a practical perspective, the ODS‐C could be utilized by educators, counselors, and other practitioners working with adolescents. The ODS‐C could inform targeted interventions designed to promote responsible online behavior while still allowing adolescents to benefit from positive aspects of digital communication.

## Conflicts of Interest

The authors declare no conflicts of interest.

## Data Availability

The data that support the findings of this study are available from the corresponding author upon reasonable request.
